# Human papillomavirus testing in cervical cancer screening

**DOI:** 10.1038/sj.bjc.6602580

**Published:** 2005-05-03

**Authors:** S Franceschi, C Mahé

**Affiliations:** 1 International Agency for Research on Cancer, 150 cours Albert Thomas, 69372 Lyon cedex 08, France

Research on the use of human papillomavirus (HPV) DNA testing in the screening and management (triage) of cervical lesions began in the late 1980s with the growing evidence that certain (high-risk (HR)) HPV types were the cause of cervical cancer (Walboomers *et al*, 1999; IARC, 2005). Since women not infected with HR HPV types, even with abnormal cytology, are at negligible risk for cervical cancer, HPV testing could be superior to cytology in cervical cancer screening (Cuzick *et al*, 1999; Nobbenhuis *et al*, 1999). Human papillomavirus testing may also be cost-effective if it allows for a longer screening interval, or for screening to be discontinued at an earlier age than currently recommended (i.e., 65 years). Follow-up studies of women with negative cytology, according to HPV status, are therefore important. In this issue, Bulkmans *et al* (2005) and Grainge *et al* (2005), using two different study designs, provide further evidence of the value of adding HPV testing to cervical cytology in screening programmes.

In their 5-year cohort study in The Netherlands of 2810 women aged 30–60 years with normal cytology, Bulkmans *et al* (2005) show that, in agreement with previous findings from the US (Sherman *et al*, 2003), France (Clavel *et al*, 2004) and the UK (Cuzick *et al*, 2003; Peto *et al*, 2004), HR HPV testing combined with cytology has higher sensitivity and higher negative predictive value for cervical intraepithelial neoplasia (CIN) 3 and cancer than cytology alone. Specificity was, however, slightly lower for HPV testing and cytology (93%) than cytology alone (95%).

The improvement in sensitivity and negative predictive value made possible by the addition of HR HPV testing to cytology supports the use of viral testing in order to increase the screening interval, although the findings of Bulkmans *et al* (2005) should be interpreted with some caution as their study was relatively small, and, as the authors emphasise, an increased sensitivity is implicit in performing any additional test (Franco and Ferenczy, 1999).

Also in this issue, Grainge *et al* (2005) report the findings of a nested case–control study based on women with a normal cytological smear between 1988 and 1992 (termed the baseline smear) who later, after an average of 6.8 years, received a histologically confirmed diagnosis of CIN 2 or worse. Excluding CIN2, which is biologically and clinically difficult to distinguish from CIN1, there were 346 CIN3 cases and 51 cervical cancer cases together with 591 control women who never had cytological abnormalities. Although Grainge *et al* (2005) found a significantly higher HPV positivity among cases compared to controls, a substantial proportion of women tested negative for HPVs in baseline smears less than 4 years (67%), and 4–13 years (74%), before the diagnosis of CIN3/cervical cancer. Unfortunately, such use of long-stored archived cervical smears is prone to the danger of false-negative HPV findings (De Roda, 1995) and crosscontamination (Chua and Hjerpe, 1995).

It is important to bear in mind, however, that the detection of HPV at a single point in time (as in case–control studies) or over a few years of follow-up (seldom more than 5 years in available cohort studies, Schiffman *et al*, 2005) does not allow an accurate estimate of a woman's lifelong risk of HPV infection nor of cervical cancer. Transiently detectable HPV infection is generally regarded as harmless, but the long-term prognosis of an HPV-positive finding is not fully understood. A follow-up study of 232 women with HR HPV infection in Manchester, UK, showed a cumulative rate of CIN3 or worse of 16% after 10 years (Peto *et al*, 2004). A lower CIN3 rate (6%) was seen in the Portland study in the US (Sherman *et al*, 2003), in which women were examined, biopsied and treated more frequently than in the Manchester study.

At least two important issues are, therefore, still open: (a) whether HPV infection disappears or, in some instances, persists undetectably in the basal cells of the cervix, and (b) whether HR HPV infection without cervical abnormality should be observed or treated, and if so, when and how. These uncertainties must be resolved before HPV testing can replace cytology and new recommendations made about screening interval or early screening cessation (Peto *et al*, 2004).

Several large-scale randomised controlled trials are ongoing on these issues and their final results should become available within the next 2 or 3 years (Dillner, 2000; Bulkmans *et al*, 2004; Kitchener *et al*, 2004; Ronco *et al*, 2004). For the moment it is important to avoid the so-called ‘implement now and trust’ approach (Whynes, 2004). Apparent coincidence of interests between manufacturers, who see an enormous market for HPV tests, and women and physicians, who would favour any extra test for added reassurance, should not distract from the evaluation of the real benefit of using HPV testing in cervical cancer screening and its associated financial and emotional cost. Most importantly, enthusiasm for new technology should not eclipse the well-known requirements for good screening programmes, namely high coverage, quality control and follow-up (IARC, 2005).
